# SERPINE2 Overexpression Is Associated with Poor Prognosis of Urothelial Carcinoma

**DOI:** 10.3390/diagnostics11101928

**Published:** 2021-10-18

**Authors:** Hao-Wen Chuang, Kan-Tai Hsia, Jia-Bin Liao, Chih-Ching Yeh, Wei-Ting Kuo, Yi-Fang Yang

**Affiliations:** 1 Department of Pathology and Laboratory Medicine, Kaohsiung Veterans General Hospital, Kaohsiung 813414, Taiwan; hwchuang1980@gmail.com (H.-W.C.); jbliao@vghks.gov.tw (J.-B.L.); ccyeh@vghks.gov.tw (C.-C.Y.); 2 Institute of Oral Biology, School of Dentistry, National Yang Ming Chiao Tung University, Taipei 11221, Taiwan; kthsia@ym.edu.tw; 3 Department of Nursing, Shu-Zen Junior College of Medicine and Management, Kaohsiung 82144, Taiwan; 4 Division of Urology, Department of Surgery, Kaohsiung Veterans General Hospital, Kaohsiung 813414, Taiwan; 5 Institute of Clinical Medicine, National Yang Ming Chiao Tung University, Taipei 11221, Taiwan; 6 Department of Medical Education and Research, Kaohsiung Veterans General Hospital, Kaohsiung 813414, Taiwan

**Keywords:** *SERPINE2*, bladder cancer, upper urinary tract cancer, urothelial carcinoma, prognosis

## Abstract

Recent studies have reported that *SERPINE2* contributes to the development of various cancers. However, its association with urothelial carcinoma (UC) remains unclear. In this study, data on urinary bladder UC (UBUC) cases from The Cancer Genome Atlas (TCGA) database were used to investigate the prognostic value of SERPINE2 mRNA expression. Then, SERPINE2 expression was analyzed with tissue microarrays constructed from 117 upper tract UC (UTUC) and 84 UBUC tissue specimens using immunohistochemical staining. Results were compared to clinicopathologic data by multivariate analysis. In the TCGA database, high SERPINE2 mRNA expression indicated a poor prognosis in patients with UBUC. Furthermore, Mann–Whitney U test showed that high SERPINE2 immunoexpression was significantly associated with adverse pathologic parameters including invasion, high grade, coexistence of UC in situ, and advanced pT stage (all *p* < 0.05, except for a marginal association with high-grade UBUC, *p* = 0.066). Kaplan–Meier analysis revealed that high SERPINE2 expression was associated with worse overall survival (OS; UTUC, *p* = 0.003; UBUC, *p* = 0.014) and disease-free survival (UTUC, *p* = 0.031; UBUC, *p* = 0.033). Moreover, multivariate analysis identified high SERPINE2 expression as an independent prognostic factor for OS (UTUC, *p* = 0.002; UBUC, *p* = 0.024). Taken together, our findings demonstrated that increased SERPINE2 expression is associated with adverse pathologic features and may serve as a prognostic biomarker for UC.

## 1. Introduction

Urothelial carcinoma (UC) is a common urinary tract malignancy worldwide, involving the lower (bladder and urethra) or upper (renal pelvis and ureter) urinary tract. Urinary bladder UC (UBUC) is the 10th most common cancer worldwide, with a yearly incidence of approximately 430,000 cases; it ranks 13th in terms of yearly mortality from cancer [1]. In contrast, upper tract UCs (UTUCs) are rare, accounting for approximately 5–10% of all urothelial malignancies, with an estimated annual incidence of two new cases per 100,000 people in Western countries [2]. Although there are various treatment modalities and advancements in UC treatment, the prognosis of patients over the past two decades remains poor [3,4,5]. Hence, the discovery of new prognostic biomarkers remains an urgent concern given the need for improving clinical outcomes in patients with UC.

Serine proteinase inhibitor clade E member 2 (SERPINE2), also known as protease nexin-1, is a member of the serine protease inhibitor superfamily and was first identified as a neurite-promoting factor for the release of cultured glioma cells [6]; various other cells such as fibroblasts, vascular smooth muscle cells, endothelial cells, platelet particles, and chondrocytes have also been reported to secrete the same [7,8,9,10]. As a serine protease inhibitor, SERPINE2 has been known for its anti–serine protease activity against thrombin, urokinase, plasminogen, and other serine proteinases [11,12,13]. Tumor cells have also been found to secrete SERPINE2, with studies showing that abnormal expression of SERPINE2 contributes to tumorigenesis and tumor invasion in various cancers, including breast cancer, pancreatic cancer, colorectal cancer, gastric cancer, testicular cancer, skin melanoma, osteosarcoma, thyroid cancer, oral squamous cell carcinoma, endometrial cancer, esophageal squamous cell carcinoma, lung adenocarcinoma, and hepatocellular cell carcinoma [8,14,15,16,17,18,19,20,21,22,23,24,25,26]. A recent study on UBUC demonstrated that *SERPINE2* might play important roles in activating and recruiting immune cells, which can significantly impact tumor behavior regulation and treatment response [27]. However, the association between SERPINE2 expression and prognosis among patients with UC remains unclear.

To address this, the present study initially performed survival analysis for UBUC using The Cancer Genome Atlas (TCGA) database. Thereafter, SERPINE2 immunoexpression in UTUC and UBUC tissues and its association with various clinicopathological features as well as patient prognosis, overall survival (OS), and disease-free survival (DFS) were investigated.

## 2. Materials and Methods

### 2.1. Survival Analysis of SERPINE2 in UBUC

We analyzed the TCGA dataset by the Gene Expression Profiling Interactive Analysis (GEPIA) tool (http://gepia.cancer-pku.cn/index.html) on 1 May 2021 [28]. The GEPIA database contained survival data on 402 patients with UBUC, which were divided into 201 patients with high-risk and 201 patients with low-risk groups based on the median, to construct the OS and DFS curves by using Kaplan–Meier analysis.

### 2.2. Patient Cohort

Our study protocol was approved by the institutional review board of Kaohsiung Veterans General Hospital (approval number: VGHKS19-CT2-06). This study included 117 patients with UTUC (57 males and 60 females; mean age 69 years, range 47–89 years) who underwent nephroureterectomy and 84 patients with UBUC (62 males and 22 females; mean age 71 years, range 26–91 years) who underwent either transurethral resection of bladder tumor or cystectomy with curative intent between 2008 and 2017 at Kaohsiung Veterans General Hospital. None of the patients had received radiation therapy or chemotherapy before surgery. The clinicopathological characteristics of the patients were obtained from medical records and follow-up data. Tumor progression was defined as the development of recurrence at the site of nephroureterectomy and lower urinary tract (e.g., the bladder), lymph node metastasis, visceral metastasis, or death. Patients were followed up from initial primary tumor resection until the appearance of any event of interest or the end of the study. Patients who did not experience any event of interest by the end of the study were censored during time-to-event analyses. OS was defined as the period from initial primary tumor resection to death or last follow-up. DFS was defined as the period from the follow-up to tumor progression, as defined earlier.

All sections were retrieved and reviewed by two experienced pathologists (H.-W.C. and J.-B.L.) to confirm the original diagnosis and stage of each case according to the 2016 World Health Organization Classification of Tumours of the Urinary System and Male Genital Organs [29] and the 8th edition of the American Joint Committee on Cancer Staging Manual [30], as well as to select representative paraffin blocks for tissue microarray (TMA) constructions (Supplemental Table S1).

### 2.3. TMA Construction

Two morphologically representative tumor areas and one benign urothelium area were selected from the cases, including 117 UTUC and 84 UBUC tissue specimens. Two tissue cylinders from the marked tumor area and one tissue cylinder from the benign urothelium area with a diameter of 1.5 mm were punched from each donor block and then brought into the recipient paraffin blocks using a precision instrument (Manual Tissue Arrayer, MTA-1, Beecher Instruments, Sun Prairie, WI, USA); then, five tissue microarrays were constructed. Subsequently, 4-um thick serial sections were cut from the TMA blocks for staining and immunohistochemical (IHC) studies (Supplemental Figure S1).

### 2.4. IHC Method 

IHC procedures were performed using the Bond III Autostainer (Leica Biosystems Newcastle Ltd., Newcastle Upon Tyne, UK) based on the manufacturer’s recommendations. In brief, tissue sections were deparaffinized and rehydrated; afterward, antigen retrieval was performed by immersing the slides in ethylenediaminetetraacetic acid buffer (pH 9.0; catalog number AR9640) for 20 min. The sections were then incubated with primary antibodies against SERPINE2 (1:100; catalog number MA5-25936; Invitrogen, Carlsbad, CA, USA) at room temperature for 30 min, a bond polymer refine detection kit (catalog number DS9800) with postprimary and polymer reagent for 8 min, 3,3-diaminobenzidine for 10 min, and hematoxylin as a counterstain for 5 min. A negative control incubated without the primary antibody was used to ensure immunostaining quality.

### 2.5. IHC Evaluation

In each case, the cytoplasm and membrane of cells stained with SERPINE2, regardless of the intensity of the stain, were counted as positive. Stained tissue sections were reviewed and scored separately by two pathologists (H.-W.C. and J.-B.L.) who had no prior knowledge regarding the clinical information on the cohort. Moreover, a consensus regarding controversial cases was achieved using a multiheaded microscope. SERPINE2 expression was scored using a semi-quantitative H-score method, which considered both the intensity of the staining and the percentage of positively stained cells. The intensity of the cytoplasmic or membranous immunostaining was scored on a 4-point scale: 0 (negative staining), 1+ (weak staining), 2+ (moderate staining), and 3+ (strong staining). The percentage of SERPINE2-immunostained cells was also evaluated using scores ranging from 0 to 100. Final scores were calculated by multiplying the two scores derived from the percentage of SERPINE2-immunostained cells and staining intensity to obtain an immunostaining score ranging from 0 to 300.

### 2.6. Statistical Analysis

SPSS software version 20.0 (SPSS Inc., Chicago, IL, USA) was used to perform statistical analyses. The difference between the two groups was compared by the paired *t*-test. Associations between SERPINE2 scores and clinicopathological characteristics, including age at diagnosis, gender, location, the status of invasion, tumor grade, the coexistence of UC in situ (UIS), primary tumor status (T stage), the extent of lymph node metastasis (N stage), and the extent of distant metastasis (M stage), were analyzed using the Mann–Whitney U test. The Kaplan–Meier method and log-rank test based on a median cut-off immunoscore value of 65 and 83 for UTUC and UBUC, respectively, were used to evaluate significant differences in OS and DFS. Univariate and multivariate analyses of survival distributions were performed using Cox proportional hazards models. All *p*-values < 0.05 indicated statistical significance.

## 3. Results

### 3.1. Survival Analysis of SERPINE2 in UBUC Using TCGA Dataset

**Figure 1:**
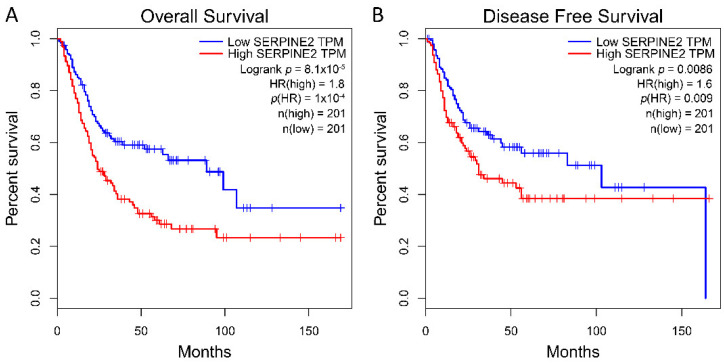
Survival analysis using TCGA database plotted using Kaplan–Meier curves. The overall survival (**A**) and disease-free survival (**B**) curves comparing patients with high (red) and low (blue) SERPINE2 mRNA expressions were plotted. The log-rank test showed that high SERPINE2 mRNA expression predicted poor overall and disease-free survivals in bladder carcinoma.

TCGA database analysis found that high SERPINE2 expression was a predictor of poor prognosis for both OS and DFS in patients with bladder carcinomas (Kaplan–Meier plots and log-rank test, *p* < 0.01 for both OS and DFS, Figure 1A,B).

### 3.2. Clinicopathological Findings for UTUC

The clinicopathological features of patients with UTUC are presented in Table 1. The disease showed a slight predilection for females. Most patients had invasive tumors (n = 90, 76.9%) and high histological grade (n = 88, 75.2%). A total of 67 patients (57.3%) presented with coexisting UC in situ (UIS). Advanced pT stage (pT2–T4) was observed in 54 patients (46.2%). Nodal metastasis was observed in 5 patients (4.3%), and distant metastasis was noted in 21 (17.9%).

### 3.3. Clinicopathological Findings for UBUC

As outlined in Table 1, patients with UBUC were predominantly male (n = 62, 73.8%) and aged >65 years (n = 62, 73.8%). A majority of the patients (n = 60, 71.4%) had high histological grade, with 25 (29.8%) patients having coexisting UIS. Advanced pT stage (pT2–T4) at initial diagnosis was observed in 26 patients (31.0%). Only four patients (4.8%) presented with lymph node metastasis, whereas 16 (19.0%) exhibited distant metastasis.

### 3.4. Association of SERPINE2 Immunoreactivity with UTUC and UBUC

**Figure 2:**
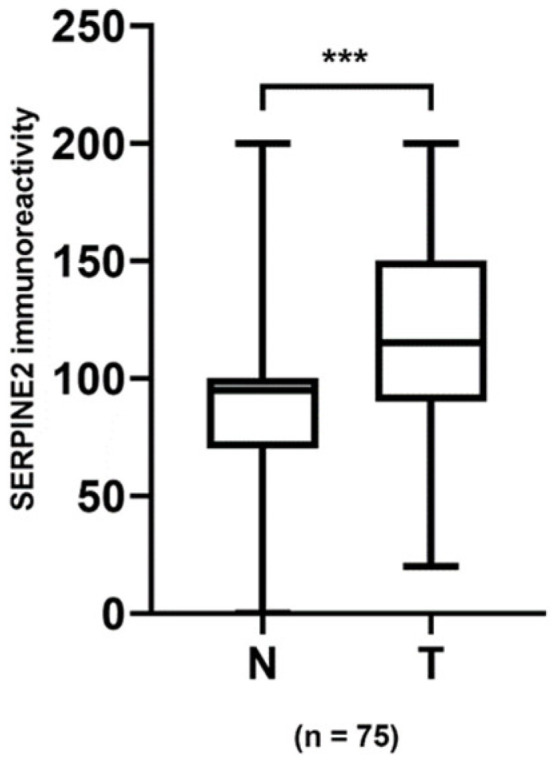
SERPINE2 was upregulated in urothelial carcinoma tissues. Quantification of SERPINE2 immunoexpression in 75 paired urothelial carcinoma specimens and significance determined by the paired *t*-test. ***, *p* < 0.001.

**Figure 3:**
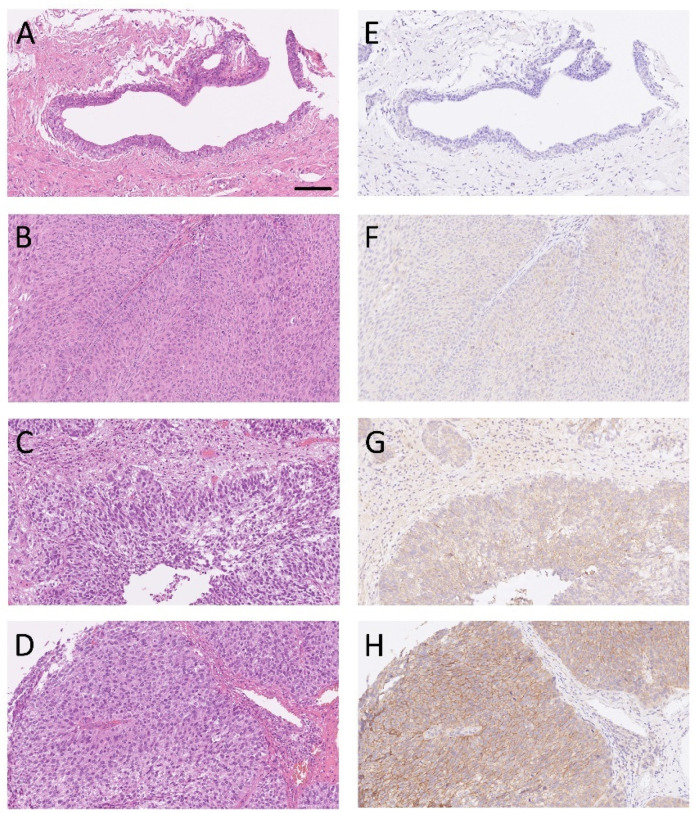
Representative sections of the normal urothelium (**A**), low-grade urothelial carcinoma (UC) (**B**), and high-grade UC (**C**,**D**) stained with H&E and immunostained for SERPINE2 (**E**–**H**). SERPINE2 immunoreactivity to the normal urothelium, low-grade UC, and high-grade UC was observed as (**E**) negative, (**F**) weak (1+), (**G**) moderate (2+), and (**H**) strong (3+). Scale bar in (**A**), 100 µm. The scale bar applies to all panels.

SERPINE2 was primarily detected on the cell membrane and cytoplasm of UTUC and UBUC cells, with varying staining intensity and distribution. Forty-seven and twenty-eight patients with UTUC and UBUC, respectively, had available normal urothelium. To examine the expression levels of SERPINE2 in urothelial carcinoma, 75 sets of tumor specimens and paired normal urothelium (47 UTUC and 28 UBUC) were analyzed by IHC. In 53 of the 75 cases, SERPINE2 showed a tendency to show greater positive expression in tumors than in normal urothelium (Figure 2, *p* < 0.001). High-grade tumors more often exhibited SERPINE2 overexpression than low-grade tumors (Figure 3A–H). As illustrated in Table 1, SERPINE2 overexpression was indeed significantly associated with histological grade in UTUC (*p* = 0.001). Although SERPINE2 expression was not significantly associated with UBUC histological grade, a tendency was still observed (*p* = 0.066). The immunoscore expressed by SERPINE2 was also significantly associated with invasion (*p* = 0.001), UIS (UTUC, *p* = 0.033; UBUC, *p* = 0.003), and advanced pT stage (UTUC, *p* < 0.001; UBUC, *p* = 0.003). Nevertheless, no significant associations with age, gender, tumor location, or lymph node, and distant metastasis were noted.

### 3.5. Survival Analysis for UTUC and UBUC

**Figure 4:**
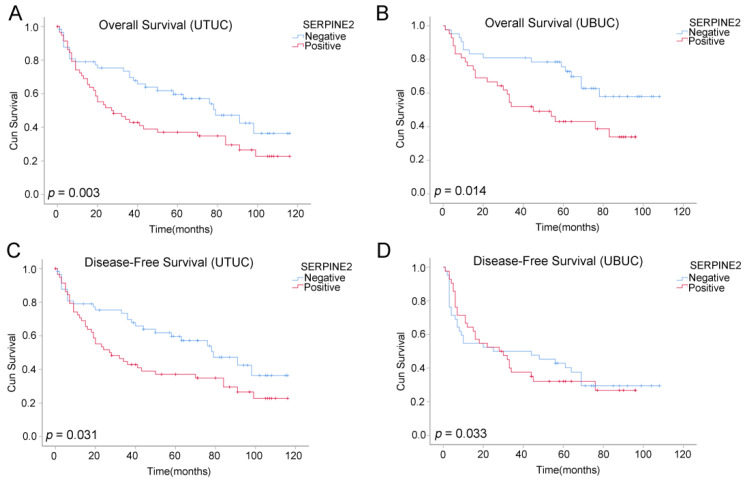
(**A**,**B**) Overall survival and (**C**,**D**) disease-free survival rates according to SERPINE2 immunoreactivity in both upper tract and bladder urothelial carcinoma.

Follow-up data over a period ranging from 1 to 120 (median, 58) months for UTUC and 1 to 108 (median, 58) months for UBUC were available for all patients. The OS and DFS curves obtained using the Kaplan–Meier method are shown in Figure 4A–D. Patients with UTUC and UBUC who had high SERPINE2 expression exhibited worse OS than those with low expression (UTUC, *p* = 0.003; UBUC, *p* = 0.014; log-rank test); similar results were observed for DFS (UTUC, *p* = 0.031; UBUC, *p* = 0.033; log-rank test). As shown in Table 2, univariate analysis for patients with UTUC revealed that age ≥ 65 years (*p* = 0.001), invasion (*p* = 0.045), high grade (*p* = 0.002), UIS (*p* = 0.011), advanced pT stage, lymph node and distant metastasis (*p* < 0.001, respectively), and high SERPINE2 immunoscore (*p* = 0.004) were significantly associated with decreased patient OS. Multivariate analysis revealed that age ≥ 65 years (*p* < 0.001), invasion (*p* = 0.008), high grade (*p* = 0.001), distant metastasis (*p* < 0.001), and high SERPINE2 immunoscore (*p* = 0.002) were independent prognostic factors for poor OS in patients with UTUC. As summarized in Table 3, univariate analysis for patients with UBUC revealed that age ≥ 65 years (*p* = 0.004), high grade (*p* = 0.044), advanced pT stage (*p* < 0.001), lymph node and distant metastasis (*p* = 0.002 and *p* < 0.001, respectively), and high SERPINE2 immunoscore (*p* = 0.016) were significantly associated with poor OS. Multivariate analyses identified age ≥ 65 years (*p* = 0.004), distant metastasis (*p* < 0.001), and high SERPINE2 immunoscore (*p* = 0.024) as significant independent prognostic factors for poor OS.

## 4. Discussion

Numerous studies have reported SERPINE2 overexpression in various cancers, which has been associated with the degree of cancer malignancy [8,14,15,16,17,18,19,20,21,22,24,25,26]. Utilizing the TCGA database, this study detected higher SERPINE2 mRNA expression in UBUC, which has been associated with poor survival. The abovementioned finding is consistent with those of a study by Huang et al. [31]. Recently, Lin et al. [32] also identified that *SERPINE2* could predict OS in patients with UBUC based on five cohorts. Our study showed that for both UTUC and UBUC, increased SERPINE2 immunoexpression was associated with poor prognostic factors, including UIS coexistence, invasion, and advanced pT stage. Significant and marginal associations between increased SERPINE2 immunoexpression and high tumor grade have also been noted for UTUC and UBUC, respectively; higher SERPINE2 immunoexpression was associated with worse OS and DFS. Furthermore, the Cox regression model identified SERPINE2 immunoexpression as a significant predictor for worse OS rates (*p* < 0.05). Taken together, the aforementioned findings indicate that *SERPINE2* plays a key role in UC development and progression, confirming the utility of SERPINE2 as an important biomarker for UC prognosis.

More patients with increased SERPINE2 immunoexpression exhibited UIS coexistence. Considering that patients who had UBUC with UIS exhibited aggressive and multifocal tumors, cystectomy was adopted even for NMIBCs, as recommended by the European Association of Urology [33]. As such, careful examination for the presence of other UC foci should be recommended in UTUC patients with increased SERPINE2 immunoexpression.

Our results showed that high-grade UTUC tumors exhibited statistically higher SERPINE2 immunoexpression than low-grade ones (*p* = 0.001). Although a significant association was not observed in UBUC (*p* = 0.066), a marginal association was still noted. The aforementioned findings are consistent with the observations in osteosarcoma [20]. Recently, a study reported that SERPINE2 expression induced by epidermal growth factor (EGF) through the EGF/MEK/ERK pathway caused cell proliferation [14]. Moreover, reports have shown that *SERPINE2* can inhibit plasminogen-induced apoptosis of Chinese hamster ovary fibroblasts, which constitutively express tissue-type plasminogen activator [34]. Consistent with previous study findings, the present study found that patients with advanced pT stage had higher SERPINE2 immunoexpression. Such results, coupled with the association between increased SERPINE2 immunoexpression and higher tumor grade, suggest that SERPINE2 overexpression is associated with the development and progression of UC malignancy, indicating its potentially significant role as an oncogene in UC.

*SERPINE2* has been shown to promote metastasis in various human cancers through several mechanisms such as tumor matrix remodeling and tumor-associated macrophage polarization [35], glycogen synthesis kinase 3β signaling pathway activation [19], P38 signaling pathway activation [36], and bone morphogenetic protein 4 expression through Wnt/β-catenin pathway activation [24]. Although the present study showed that increased SERPINE2 immunoexpression was indeed associated with invasive tumors in both UTUC and UBUC, no significant association with nodal and distant metastasis was noted. In fact, a study using prostate cancer cells reported that *SERPINE2* promoted apoptosis [9], whereas a study by Xu et al. [37] showed that SERPINE2-induced inhibition of urokinase-type plasminogen activator prevented prostate cancer cell invasion. Such findings seem to suggest that *SERPINE2* has diverse biological functions in different types of cancer. Moreover, a recent study also suggested that *SERPINE2* is an immune-related prognostic gene for UBUC [27]. Nonetheless, further in vitro, and even in vivo, studies are certainly needed to elucidate the molecular mechanisms of *SERPINE2* in UTUC and UBUC.

Urinary cytology is a simple, non-invasive, and cost-effective procedure for screening patients with suspected urothelial malignancies and for following up the patients after treatment. However, low sensitivity to low-grade lesions and equivocal results have been major limitations for this procedure. Fortunately, low-grade papillary tumors are generally detected easily by cystoscopy. However, equivocal results caused by inadequate cellularity of samples and cellular degeneration can cause management dilemmas for clinicians [38]. To overcome the aforementioned limitations, several urinary protein biomarkers, such as bladder tumor antigen and nuclear matrix protein 22, have been developed for UC detection. However, none of the urinary protein biomarkers investigated to date can be used for the accurate noninvasive detection of UC [39]. To our knowledge, no studies have so far utilized urinary SERPINE2 levels for UC detection. The association between SERPINE2 expression and high grade and invasive UC observed in our study may suggest its potential as a urinary biomarker for UC detection.

## 5. Conclusions

The present study found that SERPINE2 was highly expressed in both UTUC and UBUC and subsequently confirmed its role as an independent prognostic factor for both malignancies. Moreover, our results showed that SERPINE2 overexpression is associated with both poor OS and DFS and provided important evidence supporting the potential for SERPINE2 as a prognostic biomarker for UC. Clarifying the underlying molecular mechanisms of *SERPINE2* for UC in further studies would certainly help *SERPINE2* emerge as an attractive therapeutic target for patients with UC.

## Figures and Tables

**Table 1:** Relationships between the immunoscore of SERPINE2 and clinicopathological parameters in 117 UTUC and 84 UBUC patients.

Parameter	Upper Urinary Tract Urothelial Carcinoma				Urinary Bladder Urothelial Carcinoma			
	No.	Mean ± SEM	Median	*p*	No.	Mean ± SEM	Median	*p*
Age								
<65	53	80.36 ± 9.44	68	0.065	22	93.86 ± 15.34	83	0.558
≥65	64	58.47 ± 5.76	60		62	78.39 ± 7.58	83	
Gender								
Male	57	55.96 ± 6.40	45	0.078	62	84.27 ± 8.14	88	0.478
Female	60	76.17 ± 7.66	73		22	77.27 ± 13.02	65	
Location								
Pelvis	73	74.32 ± 7.06	70	0.083				
Ureter	44	53.07 ± 6.33	53					
Invasion								
Absent	27	36.48 ± 8.36	15	0.001 *	32	52.97 ± 8.87	38	0.001 *
Present	90	75.28 ± 5.81	70		52	100.58 ± 8.83	95	
Histological grade								
Low	29	37.41 ± 7.23	25	0.001 *	24	58.13 ± 8.158	63	0.066
High	88	75.85 ± 5.93	75		60	92.17 ± 8.78	83	
UIS								
Absent	50	54.80 ± 7.54	48	0.033 *	59	69.07 ± 7.64	70	0.003 *
Present	67	74.93 ± 6.72	75		25	114.00 ± 12.55	95	
Primary tumor status								
Ta-1	63	49.05 ± 5.86	35	<0.001 *	58	68.97 ± 7.69	68	0.003 *
T2-4	54	86.48 ± 7.83	78		26	112.50 ± 12.42	95	
Nodal metastasis								
Absent	112	64.46 ± 5.07	60	0.122	80	80.13 ± 6.92	80	0.162
Present	5	108.00 ± 32.58	95		4	128.75 ± 38.48	105	
Distant metastasis								
Absent	96	66.04 ± 5.60	60	0.921	68	80.22 ± 7.72	83	0.418
Present	21	67.62 ± 12.39	65		16	91.88 ± 15.33	83	

* Significance at *p* < 0.05; UIS, urothelial carcinoma in situ.

**Table 2:** Univariate and multivariate analysis of SERPINE2 in OS of 117 UTUC patients.

		Univariate		Multivariate	
Variables	No.	Hazard Ratio (95% CI)	*p*	Hazard Ratio (95% CI)	*p*
Age					
<65	53	Reference		Reference	
≥65	64	2.907 (1.529–5.527)	0.001 *	3.657 (1.898–7.048)	<0.001 *
Gender					
Male	57	Reference			
Female	60	1.224 (0.721–2.076)	0.454		
Location					
Pelvis	73	Reference			
Ureter	44	1.002 (0.764–1.313)	0.991		
Invasion					
Absent	27	Reference		Reference	
Present	90	2.245 (1.017–4.959)	0.045 *	0.181 (0.051–0.638)	0.008 *
Histological grade					
Low	29	Reference		Reference	
High	88	4.866 (1.759–13.459)	0.002 *	13.815 (3.092–61.720)	0.001 *
UIS					
Absent	50	Reference		Reference	
Present	67	2.088 (1.181–3.694)	0.011 *	1.294 (0.710–2.361)	0.400
Primary tumor status					
Ta-1	63	Reference		Reference	
T2-4	54	2.386 (1.379–4.127)	0.002*	1.705 (0.871–3.337)	0.120
Nodal metastasis					
Absent	112	Reference		Reference	
Present	5	6.441 (2.435–17.040)	<0.001 *	2.609 (0.739–7.153)	0.062
Distant metastasis					
Absent	96	Reference		Reference	
Present	21	3.997 (2.248–7.110)	<0.001 *	3.556 (1.892–6.687)	<0.001 *
SERPINE2 expression					
Low	58	Reference		Reference	
High	59	2.243 (1.297–3.879)	0.004 *	2.541 (1.405–4.597)	0.002 *

* Significance at *p* < 0.05; CI, confidence interval; UIS, urothelial carcinoma in situ.

**Table 3:** Univariate and multivariate analysis of SERPINE2 in OS of 84 UBUC patients.

		Univariate		Multivariate	
Variables	No.	Hazard Ratio (95% CI)	*p*	Hazard Ratio (95% CI)	*p*
Age					
<65	22	Reference		Reference	
≥65	62	4.442 (1.578–12.501)	0.005*	4.844 (1.646–14.254)	0.004 *
Gender					
Male	62	Reference			
Female	22	0.643 (0.296–1.398)	0.265		
Invasion					
Absent	32	Reference			
Present	52	1.968 (0.996–3.887)	0.051		
Histological grade					
Low	24	Reference		Reference	
High	60	2.317 (1.024–5.245)	0.044 *	1.434 (0.578–3.556)	0.437
UIS					
Absent	59	Reference			
Present	25	1.853 (0.973–3.529)	0.060		
Primary tumor status					
Ta-1	58	Reference		Reference	
T2-4	26	3.560 (1.885–6.722)	<0.001 *	1.062 (0.394–2.862)	0.905
Nodal metastasis					
Absent	80	Reference		Reference	
Present	4	5.130 (1.792–14.686)	0.002 *	1.251 (0.386–4.061)	0.709
Distant metastasis					
Absent	68	Reference		Reference	
Present	16	9.036 (4.356–18.743)	<0.001 *	8.454 (2.917–24.506)	<0.001 *
SERPINE2 expression					
Low	42	Reference		Reference	
High	42	2.203 (1.156–4.198)	0.016 *	2.301 (1.117–4.742)	0.024 *

* Significance at *p* < 0.05; CI, confidence interval; UIS, urothelial carcinoma in situ.

## Data Availability

The data used to support the findings of this study are available from the corresponding author upon request.

## Supplementary Materials

The following are available online at https://www.mdpi.com/article/10.3390/diagnostics11101928/s1, Figure S1: The representative photomicrographs of tissue microarray sections, Table S1: The summary of the pTNM stage of the cohort.
