# Supplementary material for: SERPINE2 Overexpression Is Associated with Poor Prognosis of Urothelial Carcinoma
Source: Diagnostics (Basel). 2021 Oct 18;11(10):1928. doi: 10.3390/diagnostics11101928 (PMC8535068; doi:10.3390/diagnostics11101928)
Supplement: Supplementary file 1 [file diagnostics-11-01928-s001.zip › diagnostics-1394341-supplementary.pdf]

Supplemental Materials

Supplementary Table S1. The summary of the pTNM stage of the cohort.

	UTUC (n = 117)		UBUC (n = 84)
**T stage**		T stage	
Ta	28(23.9%)	Ta	32(38.1%)
T1	35(29.9%)	T1	26(31.0%)
T2	10(8.5%)	T2	13(15.5%)
T3	40(34.2%)	T3	10(11.9%)
T4	4(3.4%)	T4	3(3.6%)
N stage		N stage	
N0	112(95.7%)	N0	80(95.2%)
N1	2(1.7%)	N1	1(1.2%)
N2	3(2.6%)	N2	1(1.2%)
		N3	2(2.4%)
M stage		M stage	
M0	96(82.1%)	M0	68(81.0%)
M1	21(17.9%)	M1	16(19.0%)

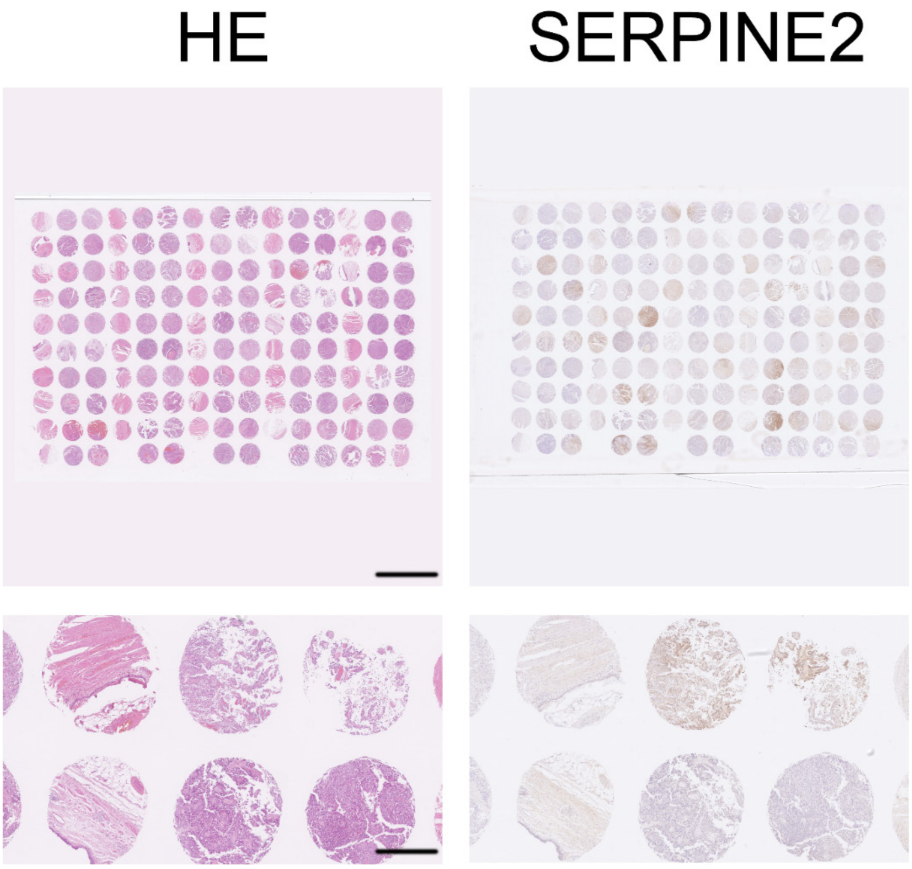

Supplementary Figure S1. The representative photomicrographs of tissue microarray sections. Scale bar in the upper panel, 5 mm; scale bar in the lower panel, 1 mm. The scale bar applies to the panels in the same row.
